# Advanced Network Sampling with Heterogeneous Multiple Chains

**DOI:** 10.3390/s21051905

**Published:** 2021-03-09

**Authors:** Jaekoo Lee, MyungKeun Yoon, Song Noh

**Affiliations:** 1 College of Computer Science, Kookmin University, Seoul 02707, Korea; jaekoo@kookmin.ac.kr (J.L.); mkyoon@kookmin.ac.kr (M.Y.); 2 Department of Information and Telecommunication Engineering, Incheon National University, Incheon 22012, Korea

**Keywords:** internet of things, sensor networks, social network services, Network (Graph) Theory, big data, large-scale network, Network (Graph) Sampling Methods, data privacy

## Abstract

Recently, researchers have paid attention to many types of huge networks such as the Internet of Things, sensor networks, social networks, and traffic networks because of their untapped potential for theoretical and practical outcomes. A major obstacle in studying large-scale networks is that their size tends to increase exponentially. In addition, access to large network databases is limited for security or physical connection reasons. In this paper, we propose a novel sampling method that works effectively for large-scale networks. The proposed approach makes multiple heterogeneous Markov chains by adjusting random-walk traits on the given network to explore the target space efficiently. This approach provides better unbiased sampling results with reduced asymptotic variance within reasonable execution time than previous random-walk-based sampling approaches. We perform various experiments on large networks databases obtained from synthesis to real–world applications. The results demonstrate that the proposed method outperforms existing network sampling methods.

## 1. Introduction

The relationship between elements in a database can be intuitively abstracted using a network-based structure, and research on networks has found practical applications that predominantly use this structure. Examples of large networks become more common in the real world: Facebook has 2.7 billion users [1], internet of things (IoT) is estimated to have 26 billion installed units by 2020, sensor networks are densely spread around the globe [2,3], metabolic networks in physiology are extremely complex [4], and Internet web pages also form a very large network [5]. Recently, network-based analysis of these databases has become increasingly important.

Network or graph analysis in biotechnology has enabled the identification of metabolic pathways [4] and new protein complexes by uncovering the various relationships among different elements [6]. Studies of social networks have shed light on how information spreads among users [7]. Electric power grids can be abstracted as a network to identify abnormal power states [8]. As the IoT and sensor networks become increasingly pervasive in our live, network analysis has been applied to mitigate emerging security issues [9]. In addition, in healthcare field, a network can analyze the spread of disease such as MERS and COVID-19 [10]. As mentioned above, study on networks has found practical applications in everyday life.

Networks or graphs requested for analysis have become larger and more complex and, subsequently, computationally quite expensive to process. Generally, network analysis algorithms have high computational complexities. For example, the time complexities for community detection using the Girvan–Newman algorithm [11], Eigenvector computation notably used for PageRank [12], and the graphlet counting algorithm for size *k* [13] are $O(|e{|}^{2}|n|)$, $O(|n{|}^{3})$, and $O(|n{|}^{k})$, respectively, where |n| and |e| represent the numbers of nodes and edges of a given network, respectively. If the amounts of nodes and edges are in billions, the space and time complexities are overwhelming for most computing machine in a research environment.

There are two complementary approaches to handle the database with extremely large network-structured data: The network can be analyzed using parallel processing methods, as in Pregel [14], GraphLab [15], and GraphX [16], or samples are taken from the network so that the analysis is possible on a machine with modest computing power. This paper presents a new sampling method and its algorithm on network.

From a common statistical point of view, inference from samples provides a fairly reasonable estimation of an entire population if many objects are selected randomly and uniformly, sufficient to represent the population. The proposed network sampling method aims to achieve unbiased samples in the overall distribution of properties on a given database. General sampling methods cannot be applied directly to sample a network because its statistical properties are interwoven with nodes and their links. Under nature of a network, the goal of sampling a network is to find the subset of the original network while preserving statistical properties.

The proposed network sampling method exploits the concept of a non-reversible random walk with an adjustment parameter, which is inspired by the momentum of hybrid Monte Carlo (HMC) [17]. The method makes heterogeneous multiple Markov chains by adjusted route traits on a network to avoid random behaviors. Experimental results demonstrated that the proposed method produces significantly improved sampling results over existing network sampling algorithms. In addition, the proposed method can keep lower asymptotic variance than typical random-walk-based sampling methods.

Our contributions can be summarized as follows:

- We propose the concept of a network sampling method with heterogeneous multiple Markov chains, which can traverse the entire target space on a database with network-structured data.
- We apply advanced non-reversible random walk on edge space as an augmented state to obtain better unbiased sampling results.
- Experiments on synthetic or real–world databases with scale–free network properties demonstrate that the proposed method can preserve the statistical characteristics of the original network-structured data.

In this paper, we propose a network graph sampling method that works effectively for large-scale networks, even given limitations to accessing network database for security reasons.

The organization of this paper is as follows: Section 1 treats the introduction along with a brief sketch of the proposed method. In Section 2, studies related to the proposed method are summarized as background. Section 3 presents the overview and detailed explanations of our proposed method in relation to the existing work discussed. Section 4 reports experimental results of the proposed sampling method applied to various datasets and discusses implications of the proposed method. Section 5 concludes the proposed method. The symbols used in this paper are listed in Table 1.

## 2. Related Work

To facilitate understanding of the proposed approach, in this section, we review well-known general sampling methods. We then present a brief overview of the theoretical properties of random-walk-based network sampling methods to explain how sampling can be performed on databases with network-based structure data.

### 2.1. Network (Graph) Sampling

In general, sampling methods are used to approximate the (usually intractable) integral or summation involved in the estimation of a distribution. Representative sampling examples include Monte Carlo (MC) sampling, importance sampling (IS), Metropolis–Hastings (MH) sampling [18], Markov chain Monte Carlo (MCMC) sampling [19], and hybrid Monte Carlo (HMC) sampling [17].

HMC adopts the concept of Hamiltonian dynamics (the *hybrid* Monte Carlo is thus also called the *Hamiltonian* Monte Carlo) in physics to the probability state-space to avoid random-walk behaviors that are exhibited by other sampling algorithms. The HMC uses momentum *m* as an auxiliary variable and its related gradient information to facilitate finding regions with higher probabilities when traversing the state-space for the original variable *x*. The HMC goes through two stages to extract samples. First, candidates for the next state are proposed through discrete approximation to Hamiltonian dynamics such as leapfrog, which generates multiple Markov chains. Next, the MH algorithm is performed for the proposed candidates, which are either accepted or rejected, to remove any bias associated with the discretization. This allows the Markov chain to explore the target distribution much more efficiently by avoiding random-walk behaviors, resulting in faster convergence[17,18,19]. A detailed account of the HMC can be found in previous studies[17,18,19].

**Figure 1:**
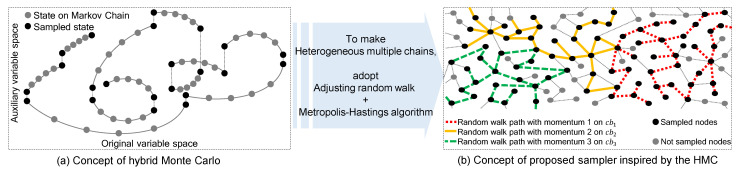
Diagrammatic explanation of what inspired the proposed network sampling method with heterogeneous multiple chains (best viewed in color).

Figure 1a depicts a toy example of several trajectories in a given state-space for a one-dimensional Gaussian distribution as the target distribution. With the heterogeneous multiple chains produced by the hybrid Monte Carlo (HMC), the gradient information by auxiliary variable space makes exploring on the space efficient and effective. The HMC makes several Markov chain trajectories in the extended space to avoid random-walk behavior. As seen in Figure 1b, we found inspiration for generating heterogeneous multiple Markov chains with transition traits within a network sampling from the HMC. This inspiration alleviates random-walk behaviors while extracting samples by creating various heterogeneous chain paths on the target space of a network.

General sampling methods have greatly influenced research on sampling large-scale networks. Representative network sampling algorithms are listed in Table 2. The main purpose of network sampling is to obtain a subset of the original network such that the statistical properties (characteristics) of the original network are well preserved without incurring excessive computational costs. Properties are the essential factors in network analysis. If a network sampling successfully preserves such properties in the extracted samples, there would be a significant reduction in computational cost compared to analyzing the original network directly. In previous network sampling studies, the quality of preserving properties was quantified by a smaller gap between the estimated distribution from the samples and the target distribution on the database.

### 2.2. Sampling under Restricted Access

In the real world, the most frequently analyzed large network-structured databases are derived from social networking services, sensor networks, and Internet—the API (application programming interface) or SDK (software development kit) of which allows only limited access to nodes and edges. Under preserving the statistical characteristics of the original network as the goal of network sampling, the proposed network sampling method is applied to extremely large databases with restricted access. In evaluating the proposed method, we assume that the database allows restricted access.

Well-known network sampling under restricted access includes traversal-based and random-walk-based sampling. These sampling methods are similar in that both extract samples by exploring the nodes on the original network in sequence; however, the existence of definite rules for selecting traversed nodes distinguishes traversal-based and random-walk-based sampling.

Common random walk on an undirected network produces finite, irreducible, and reversible Markov chains. The chain ${\left\{x_{t}\in N\right\}}_{t\geq 0}$ is *irreducible* because it is possible to transition between nodes as states and is *reversible* since a probability distribution π exists for all of states that satisfy $\pi \cdot p_{ij}=\pi \cdot Pr\left(x_{t+1}=j|x_{t}=i\right)=\pi \cdot Pr\left(x_{t+1}=i|x_{t}=j\right)=\pi \cdot p_{ji}$, which is also known as a detailed balance condition [30]. The chain is expressed as consecutive states with stationary distribution π=[d(i)/2|e|,i∈N] and transition matrix $P=[p_{ij};i,j\in N]$ [25]. In other words, a reversible Markov chain on a network obtained through a common random-walk results in an invariant distribution biased toward high-degree nodes. The properties of reversible Markov chains guarantee the irreducible condition for finite state spaces and invariant distributions.

The reversible Markov chain by random walk is used for typical network samplings such as the re-weighted random-walk sampling (RWRWS) in the importance sampling (IS) [24,25] and the Metropolis–Hastings random-walk sampling (MHRWS) derived from the Markov chain Monte Carlo (MCMC) sampling [24,25,30].

The Metropolis–Hastings (MH) algorithm is applied to both MCMC simulation for general sampling and MHRWS in the network to produce samplings [20,25]. To achieve the target distribution from samples in the network, the MH algorithm repeatedly decides whether to accept or reject a transition from the current node *i* to an adjacent node *j*. The proposal probability that affects the decision of the next node is defined as $q_{ij}=1/d\left(i\right),\;\mathrm{if}\;\left(i,j\right)\in E$. This is equivalent to the transition probability of commonly used random-walk algorithms for networks. The transition probability from node *i* to node *j* (i≠j) on a network through the MH algorithm is defined as $p_{ij}=\min\left\{1/d\left(i\right),1/d\left(j\right)\right\}=\min\left\{1,d\left(i\right)/d\left(j\right)\right\},\;\mathrm{if}\;\left(i,j\right)\in E$ or $p_{ij}=0,\;\mathrm{if}\;\left(i,j\right)\notin E$. The probability of no transition from node *i* is defined as $p_{ii}=1-\sum_{j\neq i}p_{ij}$. It is possible to produce unbiased graph sampling if the MH algorithm produces *P*, by which π generates a reversible Markov chain [20,25]. Network sampling through MHRWS requires only nodes that are connected to the current node rather than the entire network, so it can generate an unbiased sampling for networks with restricted node access [25].

Existing random-walk-based algorithms have been proposed to achieve better unbiased samples than traversal-based algorithms. However, estimation performance of these random-walk-based algorithms tends to degrade with high variance due to local region trapping and slow diffusion derived from random-walk behavior [27,31].

## 3. Proposed Method

The proposed method began with a simple question: is it possible to obtain better-quality samples from a large-scale network using random-walk-based algorithm while minimizing the drawbacks of random walk (e.g., slow diffusion over the space)? We propose a new network sampling method by imitating the concepts of the HMC [17], which offers better sampling results by avoiding random-walk behavior. The proposed method provides improved network sampling results by producing heterogenous multiple Markov chain paths to traverse efficiently the space. Under restricted access, the proposed method also achieves better unbiased sampling results than those obtained by common reversible random walk.

**Figure 2:**
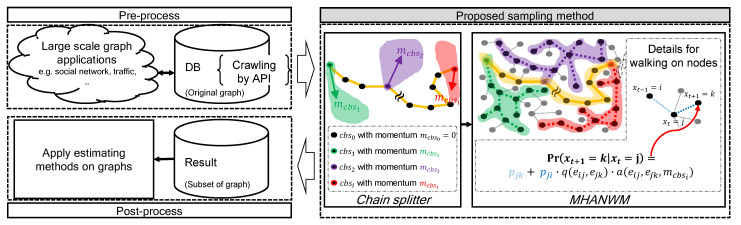
Overview of proposed method (best viewed in color).

Figure 2 shows the overall process of the proposed sampling method for estimation on a large-scale network. A key point of our work pertains to the highlighted region in gray. The proposed method produces multiple heterogenous Markov chains, which was inspired by HMC to avoid random-walk behavior. These chains are based on non-reversible random walk with different traits by adjusting an auxiliary value, similar to the movement of HMC. In addition, the diversified chains are diffused over the network by avoiding random-walk behavior.

To generate multiple heterogenous Markov chains, the newly defined momentum in the proposed method determines the movement trend between nodes while adjusting the random-walk behavior. The optimal momentum is derived from the characteristics of a scale–free network [32,33,34].

As shown in Figure 2, the proposed method consists of two major steps. First, the *chain splitter* seeks seed nodes for multiple heterogenous Markov chains and sets appropriate sample sizes for each chain. After the chain splitter step, the Metropolis–Hastings advanced non-reversible walk with momentum (MHANWM) generates each chain from the seed node with advanced non-reversible random walk with momentum parameters. This equates to dividing the entire unknown network into several chain blocks (cbs). Please note that each of cbs has a different trajectory of sequential node traversal.

### 3.1. Chain Splitter

A chain ${\left\{x_{t}\in N\right\}}_{t\geq 0}$, where *N* is the set of nodes as the original state-space, comprises consecutively visited nodes through random transition in a network. Let the previous, current, and next states be $x_{t-1}$, $x_{t}$, and $x_{t+1}$, respectively. By determining $x_{t+1}$ with $x_{t}$ and $x_{t-1}$, we can obtain a non-reversible Markov chain by avoiding backtracking. The proposed method based on a non-reversible Markov chain for reduced asymptotic variance and better convergence to a stationary distribution than a reversible chain [35,36].

In a network, the chain obtained by non-reversible random walk fails to meet the irreducibility of the Markov chain for *N* due to its dependency on previous nodes. However, the chain retains irreducibility on the augmented state-space *E*, which is made after folding the original state [30]. It is possible to easily convert the original state (*x*) to augmented state ($x^{\prime }$) in the Markov chain using $E=\{e_{ij};i,j\in N\;\mathrm{subject}\;\mathrm{to}\;Pr\left(e_{ij}\right)>0\}\subseteq N\times N$ (where |E|<∞ and $e_{ij}\neq e_{ji}$). The augmented states exploit $x_{t}^{\prime }=\left(x_{t-1},x_{t}\right)=e_{ij}\in E$ for t≥1. Augmenting the original state-space of a network such that the previous two nodes are seen through their edge maintains irreducibility. On the non-reversible chain $\left\{x_{t}^{\prime }\in E\right\}$, for a stationary distribution that is identical to the reversible chain, the asymptotic variance is less than that of its reversible chain [27,36].

With transition matrix $P=[p_{ij}]$ in the original state-space *N*, an irreducible and reversible Markov chain $\left\{x_{t}\right\}$ can be transformed to a non-reversible Markov chain $\left\{x_{t}^{\prime }\right\}$ in the augmented state-space *E* derived without backtracking. In the augmented state-space *E*, an irreducible and non-reversible chain $\left\{x_{t}^{\prime }\right\}$ from the transition matrix $P^{\prime }=\left[p\left(e_{ij},e_{kl}\right)\right]$ has a unique stationary distribution of $\pi^{\prime }\left(e_{ij}\right)=\pi \left(i\right)p_{ij}$, $e_{ij}\in E$.

The proposed method uses non-reversible random walk controlled from a momentum. The proposed method is designed to generate heterogenous multiple Markov chains with non-reversible random walk and the MH algorithm. It produces improved quality of samples compared to those produced by existing sampling methods on network-structured data under restricted access. It also incurs modest computational cost.

The chain splitter prepares sequential sampling on the network by setting seed nodes to initiate cbs with appropriate block sizes in the network. This step can be considered either sequential or parallel. In both methods, the first chain ($cbs_{0}$) is generated by non-backtracking random-walk, which tends to spread among nodes without revisiting the previous node in the network. This means that the first chain starts from an arbitrary node with irreducible and non-reversible chain $\left\{x_{t}^{\prime }\right\}$ and satisfies the following non-backtracking random-walk conditions.

$\forall e_{ij},e_{jk}\in E\;\mathrm{with}\;i\neq k\;\left(d\left(j\right)\geq 2\right),\;p\left(e_{ij},e_{jk}\right)=1/\left(d\left(j\right)-1\right)>1/d\left(j\right)=p_{jk}$, implying that $p(e_{ij},e_{ji})=0$. Here, for any node *j* with d(j)=1, $p(e_{ij},e_{ji})=0$.

After the first chain, a sequential manner can easily be considered a concatenation of various $cbs_{z},\;z\geq 0$ with their corresponding $m_{cbs_{z}}$. Therefore, the seed node for $cbs_{z}$ can be considered by the last sampled node in the previous $cbs_{z-1}$, except for the seed node in the first chain that was selected randomly. This approach is easy to implement; however, it performs not much worse than the parallel method.

In the parallel method, which was used in our experiments, the obtained first chain can be explored across the entire network. From the first chain, several hub nodes are selected as seed nodes to start the next cbs. Here, if z≥1 for $cbs_{z}$, other chains begin at the marked hub nodes of $cbs_{0}$ in parallel. It is also applicable to simple parallel implementations.

After this step, the entire network for sampling can be prepared for partitioning with several seed nodes of cbs such that multiple heterogeneous Markov chains can be generated evenly in the network. This ensures that the nodes sampled with various cbs are sufficiently covered by the original network from scattered the seed nodes. Sampled nodes can successfully capture the statistical features of the entire network. The advanced non-reversible random-walk begins from the spread seed nodes.

### 3.2. MHANWM (Metropolis–Hastings Advanced Non-Reversible Walk with Momentum)

With the preparation for $cbs_{0}$, the MHANWM performs practical sampling for the number of preassigned samples for each $cbs_{z}$ (z≥1) with its $m_{cbs_{z}}$, which is a traversal control parameter in random walk on a given network to produce various heterogenous Markov chains. During sampling within $cbs_{z}$, its $m_{cbs_{z}}$ is maintained to ensure the congruent traversal characteristics of the chain. In addition, different momentum parameters are applied to each $cbs_{z}$ for variability in the non-reversible random walk.

The characteristic of movement between nodes in each $cbs_{z}$ is defined as follows. Let the previous, current, and next nodes be *i*, *j*, and *k*, respectively, and assume that traversal is sequential with a specific momentum parameter $m_{cbs_{z}}$ for network sampling. The transition probability from node *j* to node *k* can be defined by the sum of the following two terms.

The first term refers to the transition probability to the next node *k* when the newly candidate node is not the previous node *i*. Here, k∈c(j) is proposed with 1/d(j) where $x_{t}=j\neq i\neq k$. Following the MH algorithm, a transition to the candidate node is either accepted with probability $a\left(j,k\right)={\left(d\left(j\right)/d\left(k\right)\right)}^{m_{cbs_{z}}}$ or rejected with probability 1−a(j,k), which means $x_{t+1}=j$. Thus, the probability of an accepted transition to node *k* or $x_{t+1}=k$ (when k≠i) is defined as $p_{jk}=q_{jk}\cdot a\left(j,k\right)$.

The second term indicates the transition probability with mitigated non-backtracking by momentum as a constraint parameter when the candidate node is node *k*. This is expressed as follows.

(1) $$\begin{array}{cc} Pr & \left(x_{t+1}=k|x_{t}=j,x_{t-1}=i\right)=\frac{{\left\{d\left(j\right)/d\left(k\right)\right\}}^{m_{cbs_{z}}}}{d(j)}+p_{ji}\cdot q\left(e_{ij},e_{jk}\right)\cdot a\left(e_{ij},e_{jk},m_{cbs_{z}}\right) \end{array}$$

where

(2) $$\begin{array}{cc} q(e_{ij},e_{jk}) & =1/(d(j)-1)\;\mathrm{with}\;i\neq k\;\mathrm{and}\;d(j)\leq 2 \end{array}$$

(3) $$\begin{array}{cc} a(e_{ij},e_{jk},m_{cbs_{z}}) & =\min{\left\{1,\frac{\min(1/d{\left(j\right)}^{2},1/d{\left(k\right)}^{2})}{\min(1/d{\left(j\right)}^{2},1/d{\left(i\right)}^{2})}\right\}}^{m_{cbs_{z}}} \end{array}$$

and $m_{cbs_{z}}$ lies between 0 and 1. Here, candidate nodes are adjusted to reduce bias with $d{\left(j\right)}^{\left(m_{cbs_{z}}-1\right)}$. For the momentum, we obtained an empirical $m_{cbs_{z}}$ from experiments on synthetic and real–world network datasets of scale–free network [32,33,34] to produce multiple heterogeneous chains using advanced non-reversible random walk.

If the candidate node for next node *k* is the previous node *i*, the transition is delayed, and another candidate node is proposed with the transition probability to avoid backtracking. The transition to the re-selected new node is accepted with the following probability:

(4) $$\begin{array}{c} a\left(e_{ij},e_{jk},m_{cbs_{z}}\right)=\min{\left\{1,\min\left\{1,{\left(\frac{d(j)}{d(l)}\right)}^{2}\right\}\cdot \max\left\{1,{\left(\frac{d(k)}{d(j)}\right)}^{2}\right\}\right\}}^{m_{cbs_{z}}} \end{array}$$

which is specified from Equation (3).

Thus, the transition from node *j* to node k≠i in the proposed method has probability ${\left\{d\left(j\right)/d\left(k\right)\right\}}^{m_{cbs_{z}}}/d(j)+p_{ji}\cdot q\left(e_{ij},e_{jk}\right)\cdot a\left(e_{ij},e_{jk},m_{cbs_{z}}\right)$, which is greater than the probability in the existing common reversible random-walk method.

By repeating the process of accepting and rejecting candidate nodes with the probability defined above, the accepted nodes construct chains with a heterogeneous moving pattern that is adjusted by various $m_{cbs_{z}}$. Due to the diverse trajectories of these chains, they have less correlation, and the samples obtained on the chains can be spread evenly. This approach addresses slow diffusion, local region trapping, and local looping issues by efficiently and effectively traversing the network.

The proposed method guarantees better sampling results than those obtained by common reversible random walk. These samples also approach a stationary distribution more quickly due to the non-reversible Markov chain property [35]; thus, the burn–in period is shorter than that in common random-walk-based methods.

Algorithm 1 is the pseudocode for the proposed method. Here, in Lines 1–3, the size of cbs is allocated, and a chain is created by walking among nodes repeatedly with the proposed transition probability is the chain splitter step. In Lines 5–22, the MHANWM step is performed on each $cbs_{z}$ with different $m_{cbs_{z}}$ to select a newly sampled node, and Line 23 re-weights the sampled node.
**Algorithm 1:** Network sampling at *x_t_*
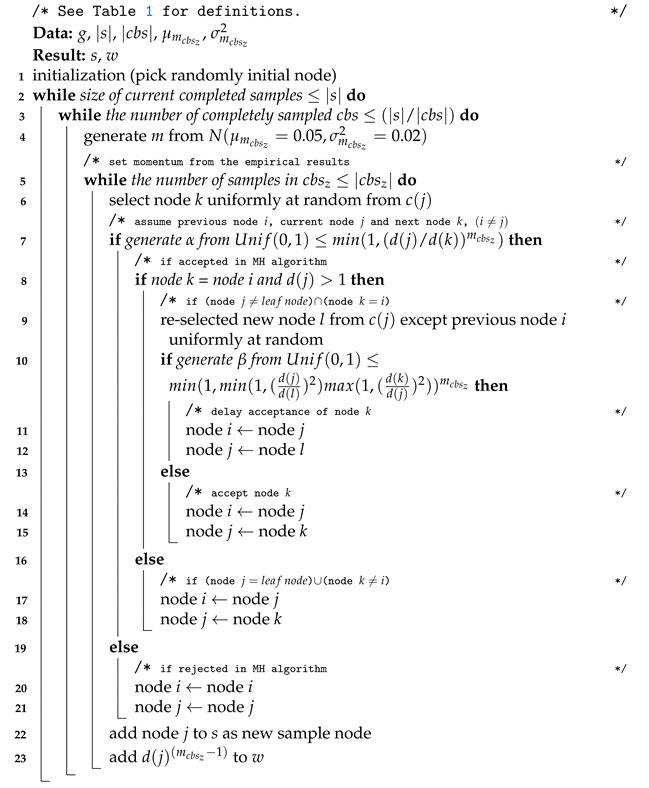



## 4. Experimental Evaluation

This section reports the experimental results of applying the proposed network sampling method to synthetic and real–world network databases, which were well-known publicly available datasets [37,38]. In the experiments, we attempted to identify network characteristics according to the proposed method. The parameters of the proposed method were tuned experimentally on given networks.

**Figure 3:**
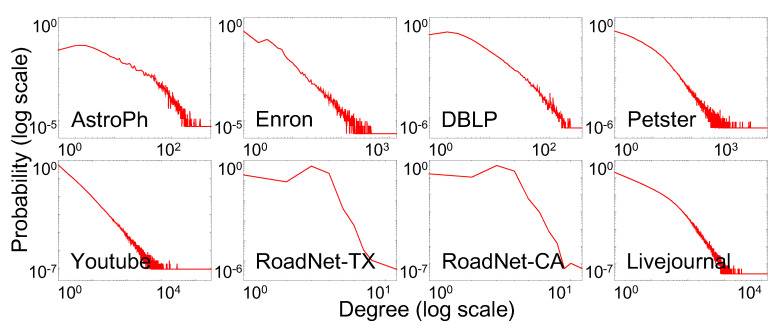
Degree distributions of real–world datasets.

These experiments were performed under the assumption of limited access on database at one time. For input, we used huge synthetic networks and eight different, large-scale real–world network databases from social networks, traffic networks, and collaboration networks. The databases are detailed in Table 3 and Figure 3. We performed all experiments on a single machine with a 3.2 GHz CPU and 16 GB RAM.

### 4.1. Evaluation Methodology

There is a wide variety of measurement methods for the quality of network sampling. Here, we compared the qualities of the sampled nodes regarding the primary network characteristics for preserving the statistical features. This evaluation method is widely used in the literature [20,21,27,31,39]. We evaluated the performance of network sampling methods by comparing the estimated distribution produced by samples and the original distribution on the statistical characteristics [20,21,27,31,39].

Well-known measuring methods for the distance between the estimated distribution from the samples and original distribution are the normalized root mean square error, which is defined as $\sqrt{E\{{\left(\hat{x}-x\right)}^{2}\}}/x$, where $\hat{x}\left(t\right)$ is the estimated value from the sample and x is a real value [40]. Similarly, the total variance distance (TVD) [41], is defined as $\left(1/2\right)\sum_{x\in N}\left|Pr\left(\hat{x}\right)-Pr\left(x\right)\right|$. In addition, the Kolmogorov–Smirnov D-statistic (KSD) [42] test quantifies the distance between the empirical distribution function of the sample and the cumulative distribution of the reference distribution. We used both metrics in our experiments.

### 4.2. Experimental Results

Many real–world networks or graphs have been reported to be scale–free network [32,33,34]. A scale–free network follows a power-law degree distribution. This means that there are many nodes with only a few links and a few nodes with many links as a hub. The probability distribution function P(k) of degree *k* of a scale–free network is described by $P\left(k\right)∼C\cdot k^{-\gamma }$ where *C* is constant, which is determined by the normalization condition, and γ called the scale–free exponent parameter whose value is typically in the range 1<γ<4, although occasionally it may lie outside these bounds [32,33,34].

Therefore, we performed the first experiment on synthetic databases with the property of scale–free network to identify optimal parameter values. To evaluate random-walk’s behavior on a scale–free network, we generated huge synthetic networks with a common scale of γ and repeated experiments by changing parameters on various γ. In this experiment, the Barab$\overset{´}{a}$si-Albert model [32], a well-known scale–free network generating model, was used to generate huge synthesized networks.

**Figure 4:**
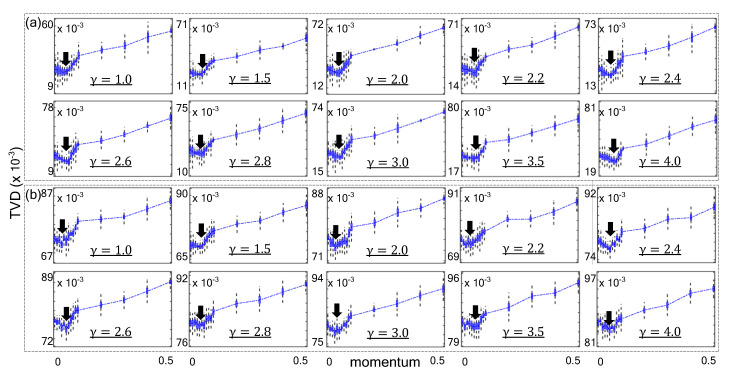
Results of sampling with various momentum parameters on huge synthetic scale–free networks with γ. (**a**) Synthetic networks with |n|=50,000,000 (**b**) |n|=100,000,000.

As shown in Figure 4, the momentum value was varied to demonstrate how TVD scores are affected by momentum. In repeated experiments, the γ of $P\left(k\right)\;C\cdot k^{-\gamma }$ for scale–free network was varied from 1 to 4. Boxplots are used to show the average and deviation. The boxplots empirically demonstrate that the proposed method obtained optimal sampling quality on synthetic scale–free networks with typical range of γ. Each minimum average TVD score is indicated with an arrow for each experiment. The proposed method minimized the TVD value when *m* was around 0.05 in the synthesized networks. We performed same experiment with real–world scale–free network databases.

**Figure 5:**
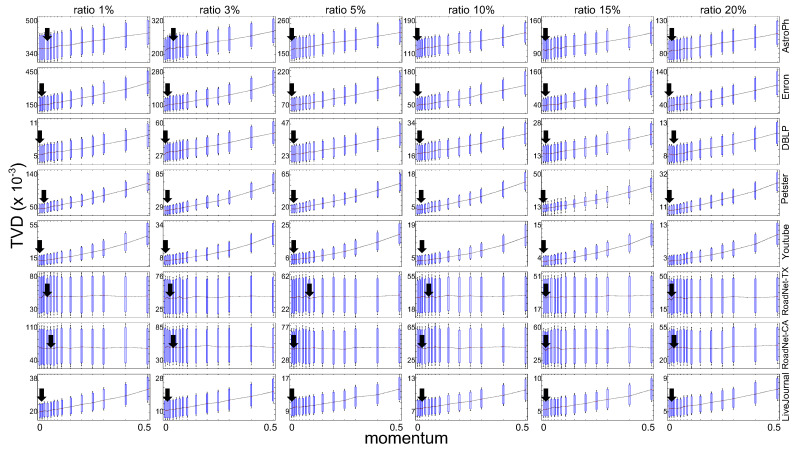
Sampling results obtained with various momentum parameters on real–world network databases.

Similar to the experiments on synthetic databases, we also monitored sampling performance to confirm a common characteristic of the proposed sampling method on real–world networks with power-law degree distribution. Figure 5 shows the performance of the proposed network sampling method while *m* was varied on real–world databases. The chart shows network sampling quality based on variations in momentum for different databases.

In experiments on real–world databases, the sampling ratios (=|s|/|n|) were set to 1%, 3%, 5%, 10%, 15%, and 20%. Other parameters were set as follows: |cbs|=1, while momentum was varied to control non-backtracking random walk. The TVD scores approached the minimum values where $\mu_{m}$ was the optimal parameter value on scale–free networks.

The results indicate that the minimum TVD score was obtained with m=0.05, where the quality of sampling would be best. As a result, we empirically confined m∼N(0.05,0.02) as the optimal momentum on scale–free networks.

**Figure 6:**
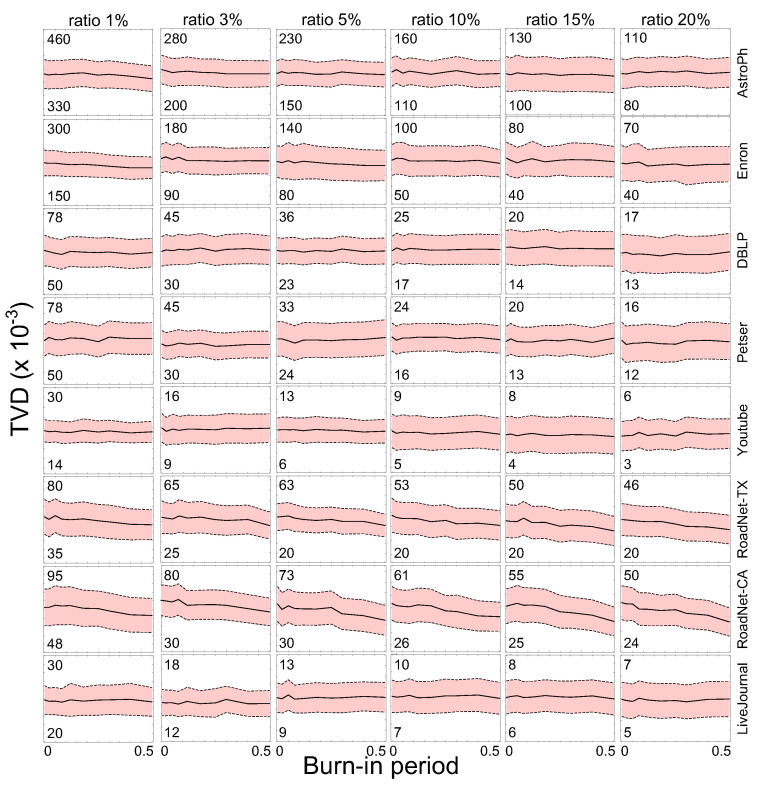
Results of sampling performance for various burn–in periods.

We also analyzed the performance of burn–in period, which is an indirect indicator of how quickly and successfully the proposed method can reach a stationary distribution. The details of this experiment are shown in Figure 6. In this experiment, the sample size and proportion of the burn–in period for each network database was varied. As can be seen, the sampling results obtained by the proposed method have little influence on the TVD scores while the burn–in periods vary. This implies that the proposed algorithm provides a stability of network sampling.

**Figure 7:**
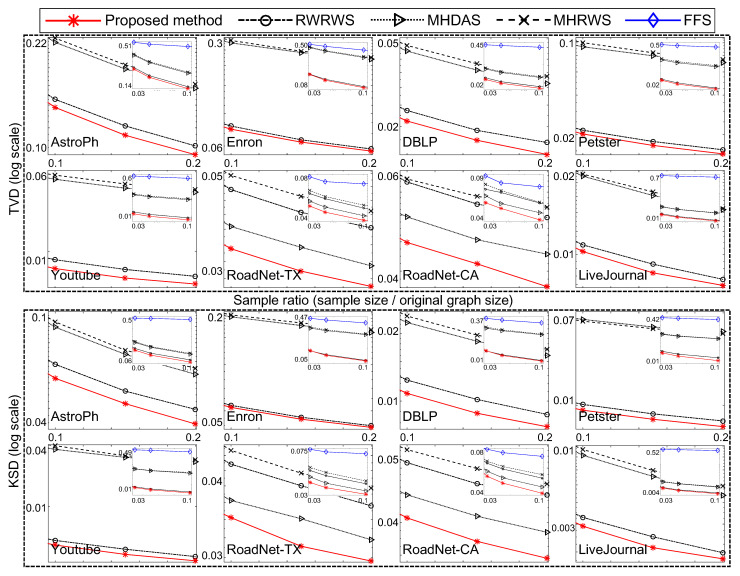
Comparison of proposed and well-known network sampling methods relative to node degree.

**Figure 8:**
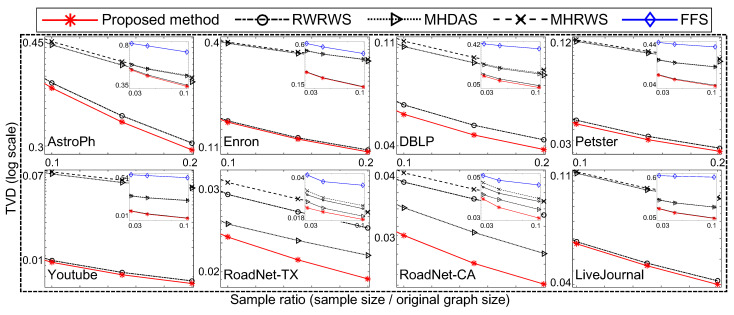
Comparison of sampling methods on clustering coefficients of networks.

In the following, we compare the sampling quality for the well-known traversal-based algorithm (i.e., FFS [21]) and random-walk-based algorithms (i.e., RWRWS [24,25], MHRWS [24,25], and MHDAS [26,27]) to the proposed method. Figure 7 and Figure 8 compare the proposed algorithm to state of the art network sampling methods.

In Figure 7, the proposed method used m∼N(0.05,0.02) and |cbs|=5. The other conditions were equal for all compared sampling methods (the FFS, RWRWS, MHRWS, MHDAS, and proposed method). For each network database, the sampling ratio was varied; and the obtained TVD and KDS results are shown for each sampling method. The proposed method obtained the lowest TVD and KDS scores for all sizes of samples, indicating it provides superior performance.

As shown, the proposed network sampling method obtained the lowest TVD and KDS scores under equal conditions. Thus, we conclude that the proposed method demonstrates superior network sampling performance with unbiased sampling on real–world network databases.

### 4.3. Discussion

**Figure 9:**
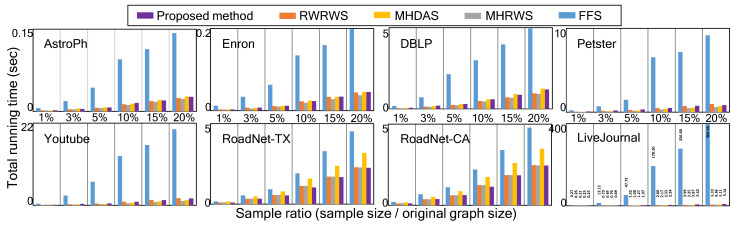
Comparison of total run time of sampling methods (colored).

The sequential version of the proposed network sampling method has a time complexity of O(|s|) and space complexity of O(|s|+|w|), where |s| and |w| are the size of the sample and its weight vector, respectively. As shown in Figure 9, the time costs of the sequential version of the proposed method are moderate, even for large network databases obtained from real applications. The run time is shown as the average of the time of 2000 independent sampling tests on each network database.

To handle extremely large network databases with limited access effectively, we can easily extend the proposed method by generating multiple cbs in parallel, as outlined in Algorithm 2. First, $cbs_{0}$ is processed as in Algorithm 1 with $m_{cbs_{0}}=0$. Then, differing from Algorithm 1, as many as the hub nodes |cbs|−1 are selected in the descending order of d(i) from the generated $cbs_{0}$. Finally, other cbs ($cbs_{z},\;z>0$) begin simultaneously from each hub as a seed node to realize parallelized sampling. From a scalability perspective, the proposed method can be also executed on existing distributed systems to analyze of huge networks.
**Algorithm 2:** Parallelization
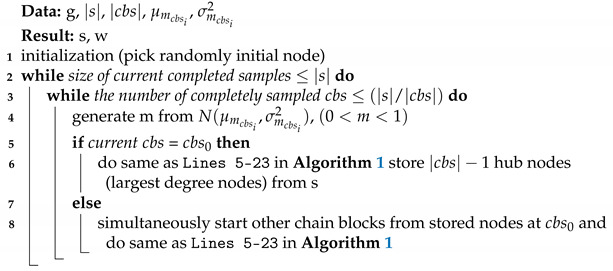


## 5. Conclusions

In this paper, we have proposed a network sampling method for databases with huge scale–free networks with improved performance on restricted access. The proposed method begins with the concept of reducing random-walk behaviors of network sampling methods by imitating multiple chains of the HMC. The proposed method adopts a momentum parameter on non-reversible random walk for a variety of state-space traversals. Multiple heterogenous Markov chains generated by the proposed method have optimized characteristics for a scale–free network. As a result, the proposed method produces effective and efficient sampling from a network database. In experiments with synthetic and real–world network databases, we observed and verified that the proposed method produces better unbiased samples in reasonable execution time than existing methods. In addition, the sequential and parallel versions of the proposed method can be implemented easily and are applicable to large network databases from a diverse range of practical applications such as the Internet of Things, sensor networks, social networks, and traffic networks. 

## Figures and Tables

**Table 1:** Notations.

Notation	Definition
*g*	graph or network
*n*	node
|n|	number of nodes
*e*, $e_{ij}$	edge, edge between node *i* and node *j*
|e|	number of edges
d(i)	degree of node *i*
c(i)	neighbors of node *i*
*s*	sample set
|s|	size of total sample set
*w*	weight vector
|w|	length of weight vector
$cbs_{i}$	$i^{\mathrm{th}}$ chain block subset of total sample
|cbs|	number of chain block
*N*	original state-space
*E*	augmented state-space
*x*	original state (variable)
$x^{\prime }$	augmented state (variable)
$m_{cbs_{i}}$	momentum of $i^{\mathrm{th}}$ chain block
$\mu_{m}$	mean of the momentum distribution
$\sigma_{m}^{2}$	variance of the momentum distribution
Pr	probability
*q*	proposal probability distribution
*a*	acceptance probability
π	stationary distribution
P	transition matrix with elements $p_{ij}$
$p_{ij}$	transition probability from state $x_{t}=i$ to state $x_{t+1}=j$, $Pr(x_{t+1}=j|x_{t}=i)$
$P^{\prime }$	transition matrix of augmented state-space

**Table 2:** Representative network sampling algorithms.

Access Types	Sampling Approaches	Algorithms
Full	Node	Random Node Sampling (RNS) [20,21]
		Random Degree Node Sampling (RDNS) [21]
	Edge	Edge Random Edge Sampling (RES) [20,21]
	Node-Edge	Random Node-Edge Sampling (RNES) [21]
Full or Restricted	Traversal	Breadth First Sampling (BFS) [22]
		Depth First Sampling (DFS) [22]
		Snowball Sampling (SBS) [23]
		Forest Fire Sampling (FFS) [21]
	Random Walk	Basic Random-Walk Sampling (RWS) [21]
		Re-Weighted Random-Walk Sampling (RWRWS) [24,25]
		Metropolis–Hastings Random-Walk Sampling (MHRWS) [24,25]
		Metropolis–Hastings Random-Walk with Delay acceptance Sampling (MHDAS) [26,27]
		Random Walk with Restart Sampling (RWRS) [21]
		Random Walk with Random Jump Sampling (RWRJS) [21,28]
Stream	Online	Random Reservoir Sampling (RRS) [29]

**Table 3:** Real–world network datasets used in our experiments.

	|n|	|e|	|n| in LWCC	ACC	|triangles|	$\max_{\left(u,v\right)}$ d(u,v)
AstroPh	18,772	198,110	17,903(0.954)	0.6306	1,351,441	14
Enron	36,692	183,831	33,696(0.918)	0.4970	727,044	11
DBLP	317,080	1,049,866	317,080(1.000)	0.6324	2,224,385	21
Petster	623,766	15,699,276	601,213(0.964)	0.0284	656,390,451	15
YouTube	1,134,890	2,987,624	1,134,890(1.000)	0.0808	3,056,386	20
RoadNet-TX	1,379,917	1,921,660	1,351,137(0.979)	0.0470	82,869	1054
RoadNet-CA	1,965,206	2,766,607	1,957,027(0.996)	0.0464	120,676	849
LiveJournal	3,997,962	34,681,189	3,997,962(1.000)	0.2843	177,820,130	17

LWCC: largest weakly connected component; ACC: average clustering coefficient; $\max_{\left(u,v\right)}$d(u,v): diameter (longest shortest path between u and v). AstroPh: collaboration network of Arxiv astrophysics; Enron: email communication network from Enron; DBLP: DBLP collaboration network; Petster: family links from dog and cat social website; YouTube: YouTube online social network; RoadNet-TX: road network in Texas; RoadNet-CA: road network in California; LiveJournal: LiveJournal online social network.

## Data Availability

This paper did not generate research data to share.
